# MicroRNAs in neural crest development and neurocristopathies

**DOI:** 10.1042/BST20210828

**Published:** 2022-04-06

**Authors:** Marco Antonaci, Grant N. Wheeler

**Affiliations:** School of Biological Sciences, University of East Anglia, Norwich Research Park, Norwich NR7 7TJ, U.K.

**Keywords:** microRNA, neural crest cells, neurocristopathies

## Abstract

The neural crest (NC) is a vertebrate-specific migratory population of multipotent stem cells that originate during late gastrulation in the region between the neural and non-neural ectoderm. This population of cells give rise to a range of derivatives, such as melanocytes, neurons, chondrocytes, chromaffin cells, and osteoblasts. Because of this, failure of NC development can cause a variety of pathologies, often syndromic, that are globally called neurocristopathies. Many genes are known to be involved in NC development, but not all of them have been identified. In recent years, attention has moved from protein-coding genes to non-coding genes, such as microRNAs (miRNA). There is increasing evidence that these non-coding RNAs are playing roles during embryogenesis by regulating the expression of protein-coding genes. In this review, we give an introduction to miRNAs in general and then focus on some miRNAs that may be involved in NC development and neurocristopathies. This new direction of research will give geneticists, clinicians, and molecular biologists more tools to help patients affected by neurocristopathies, as well as broadening our understanding of NC biology.

## Introduction

Since their discovery in 1993, miRNAs have been associated with a number of physiological and pathological processes [1]. These small RNA molecules (∼22 nt) comprise ∼1–5% of the RNA species in cells but, as a single miRNA can target many genes and many genes can be targeted by multiple miRNAs, it has been estimated that ∼30% of human genes are regulated by miRNAs. As the regulation operated by these elements is subtle and can be subject to additive effects when more than one miRNA is targeting the same mRNA, it is not surprising that these elements have been mainly associated with highly regulated biological processes such as development. In recent years, more and more evidence is pointing in this direction, but many gaps still need to be filled. In particular, more effort is needed in order to include miRNAs in the gene regulatory networks (GRN) that orchestrate the development of different tissues. Together with evidence that links miRNAs and pathological conditions such as cancer and cardiac diseases, a broader understanding of their role in biological processes is required. In this review, we give an insight of what is known about miRNAs during different steps of NC development. We also stress the reason why such knowledge could be beneficial for people affected by diseases of the NC, so-called neurocristopathies (NCP), and for the clinicians treating them. Finally, we suggest further investigations in this direction can be applicable to other aspects of biology, including cancer biology, and might help the development of new innovative drugs for treating these conditions.

## Neural crest

The Neural Crest (NC), sometimes referred as the ‘fourth germ layer', is a multipotent population of cells that originate in the region between the neural and non-neural ectoderm of the developing embryo as the neural plate develops [2,3]. The NC is specific to vertebrates and required for the formation of an astonishing number of cells and tissues, such as the craniofacial skeleton, dentine in the teeth, chondrocytes, cardiac septa, the peripheral nervous system, adrenal medulla, and pigment cells [4–6].

To allow the formation of the NC, a complex GRN of secreted growth factors and transcription factors is required. NC formation starts with neural induction, a process orchestrated by a gradient of BMP signalling. Additional signalling by Wnt leads to the transcription of specifiers for the neural plate border (NPB). FGF and Notch signalling are also involved in the expression of NPB specifiers, although these two factors play different roles among various species [2,7–9].

The combined action of these signalling pathways leads to the expression of NPB specifiers, including (but not limited to): *Pax3/7*, *Zic1*, *nMyc*, *Tfap2* and *Dlx5/6* [2,7,10,11]. A precise balance between neural and non-neural ectoderm, at this stage, is fundamental for the correct formation of NC tissue [2,12]. The next step leading to the formation of NC tissue is the expression of NC specifiers such as *FoxD3* and *Snai1/2*. Their expression is enabled by the action of the NPB specifiers [2,9,13].

One of the most extraordinary properties of NC cells is their ability to undergo an epithelial to mesenchymal transition (EMT), a property that allows neural crest cells (NCCs) to migrate throughout the developing embryo. EMT requires two steps: delamination and dispersion. Initially, NCCs detach from the neural tube, they then separate from each other to start their migration to the rest of the embryo in a coordinated manner [5,14,15]. To allow the movement of NCCs through the embryo, it is necessary to modulate the activity of cell adhesion molecules, metalloproteinases and the extracellular matrix [2,13,16].

It is important to emphasise that not all the actors involved in NC development have been discovered, and that the GRN that orchestrates this process is constantly being updated [2,3,17]. In addition, other gene regulatory mechanisms, such as the regulation operated by non-coding RNA, need to be considered.

## MicroRNAs

MicroRNAs (miRNAs) are short RNA molecules of ∼22 nt involved in the post-transcriptional control of gene regulation. They act mainly as repressors of gene expression by binding to the 3′ UTR of targeted mRNAs and either cause stalling of the ribosome, or directly promote degradation of the targeted mRNAs [18].

MiRNAs were first discovered in *C. elegans* by Lee and colleagues in 1993 [1]. Since then, an increasing number of miRNAs have been characterised, together with evidence of their important roles in regulating gene expression.

The synthesis of miRNAs has been well covered in other reviews [19]. It starts with the action of RNA-Polymerase II, which transcribes a longer primary transcript, called a pri-miRNA. In most cases, the pri-miRNA has stem loops that are recognised by the RNase III, DROSHA, which together with DGCR8, cleaves the pri-miRNA and generates a smaller product of ∼70 nt. This RNA molecule, called the pre-miRNA, is exported to the cytoplasm by the action of Exportin 5. Here, the pre-miRNA is cleaved again by another RNase III, Dicer, which generates a double stranded RNA molecule of ∼22 nt. Generally, only one strand of RNA is used as mature miRNA, while the other is degraded [20].

The mature miRNA is loaded into the RNA-induced silencing complex (RISC), which is then guided to mRNA targets and allows pairing between the ‘seed' sequence of the miRNA (∼7 nt) and the 3′ UTR of the target mRNA. This pairing leads to reduced expression by two mechanisms: the removal of the poly-A tail of the mRNA and the subsequent degradation by exonuclease activity, or the blocking of translation by stalling the ribosome. The stalled ribosome then moves to subcellular organelles called P-bodies. Here, the complex can either be stored or degraded [21–24].

Although this is the main mechanism of biosynthesis of miRNAs, other non-canonical mechanisms are known. For example, Dicer-independent processing of miRNAs can occur using short hairpin RNAs (shRNAs) as substrate. In this case AGO2 completes their maturation instead of Dicer [25]. Other non-canonical mechanisms of miRNA biogenesis occur with miRNAs located in introns of transcribed genes (mirtrons) that can be produced during the splicing process and exported by Exportin 5 [26]. Another example is the methylation of the guanosine in position 7 of the capped pre-miRNA, or ‘7-methylguanosine capped pre-miRNA' [27]. This post-transcriptional modification allows the miRNA to be exported directly to the cytoplasm by Exportin 1. In both cases (mirtrons and 7-methylguanosine capped pre-miRNA), the nascent pre-miRNA is not processed by DROSHA/DGCR8.

A different mechanism of action of miRNAs is the so-called RNA activation (RNAa). RNAa is a pathway that promotes the synthesis of mRNA, instead of repressing the translation. This mechanism is mediated by AGO1 that, once it loads the miRNA, is internalised in the nucleus and binds the promoter of specific genes, using the miRNA as guide. This mechanism creates a DNA–RNA duplex (R-loop) in proximity of the promoter that enhances the binding of the RNA-Polymerase II to the DNA [28].

It is important to note that many different miRNAs can target one specific mRNA and specific miRNAs can target more than one mRNA. This mechanism allows for the intricate regulation of gene expression, in particular the presence of more than one miRNA on a single mRNA could generate a stronger silencing effect [18].

## The role of miRNAs in NC development

A role for miRNAs during development was first noted in 2003, when Bernstein and colleagues deleted Dicer in mice, observing early embryonic lethality [29]. Other groups have gone on to show miRNAs to be important in many developmental processes, such as, for example, muscle development [30].

**Figure 1.**
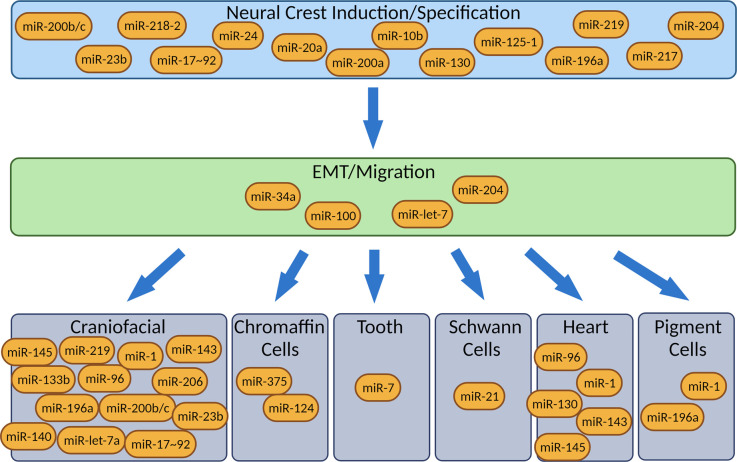
MiRNAs described in this review and where they act during Neural Crest development, from induction to differentiation.

MiRNAs associated with the NC have previously been reviewed by Weiner and colleagues [31]. Here we summarise what is known and look at more recent literature (see Figure 1 and Table 1). A role for miRNAs in NC development was originally shown by knocking out and knocking down Dicer in mice, thus disrupting miRNA biogenesis. It was observed that a NC-specific knockout of Dicer using a Wnt1-CRE is lethal [32]. This effect was due to extensive NC cell death that led to the absence of NC-derived tissues [33]. Similar effects were observed following NC-specific inactivation of DGCR8, an important co-factor of the endonuclease DROSHA. In addition, cardiac defects associated with a defect in the NC were noted [34]. It is important to note that DGCR8 is one of the genes deleted in DiGeorge Syndrome, a Neurocristopathy (NCP) that affects 1 : 4000 children [35].

**Table 1.** Neurocristopathies and associated miRNAs, with possible implicated targets

Neurocristopathy	Symptoms	miRNA or miRNA-related genes	Implicated target/s	Involvement in NC development	Reference
CHARGE Syndrome	Coloboma; Heart defects; Atresia choanae; Growth retardation; Genital abnormalities; Ear abnormalities	let-7	*chd7*	Chondrocyte differentiation	[66]
Cleft palate	Incomplete fusion of the bilateral palatal shelves	miR-140	*pdgfra*	Chondrocyte differentiation	[67]
		miR-17∼92	*tbx1, tbx3*	Induction, chondrocyte differentiation	
		miR-200b	*smad2, snai2, zeb1, zeb2*	Specification	
Congenital central hypoventilation syndrome	CNS development delay;	miR-204	*phox2b*	Specification and migration	[58]
	Hypoxic crisis				
Craniosynostosis	Premature fusion of two or more skull bones	miR-23b	*smad3, smad5*	Induction	[68]
		miR-133b	*egfr, fgfr1*	Chondrocyte differentiation	
Hirschsprung Disease	Swollen belly;	miR-100	*ednrb*	EMT [69]	[70]
	Vomiting;	miR-206	*sdpr*	Orofacial development	[63]
	Chronic constipation;	miR-214	*plagl2*		[21]
	Fatigue	miR-483	*fhl1*		[71]
		miR-124	*sox9*	Sympathoadrenal development	[72]
DiGeorge Syndrome	Behaviour problems;	DGCR8	iRNA pathway	General	[73]
	Hearing problems;				
	Feeding problems;				
	Congenital heart defects;				
	Hypoparathyroidism				
Melanoma	Skin tumours	miR-32	*mcl1*		[50]
		miR-579-3p	*mitf, braf, mdm2* (predicted)		
		miR-200c	*bmi1, zeb2, tubb3, abcg5, mdr1*	Specification	
		miR-7	*egfr, igf1r, craf*	Tooth development	
		miR-21	*pten*	Schwann cells differentiation [74]	
		miR-638	*tp53inp2*		
		miR-34a	*bcl2, cdk6, e2f3, mycn*	EMT	
		miR-100	*trim71*	EMT [69]	
		miR-125b	*bak1, bcl2, e2f3*		
		miR-192	*zeb2*		
		miR-193b	*cdk6, mcl1, bmi1* (predicted)	Orofacial development	
		miR-514a	*nf1*		
Neuroblastoma	Sympathetic nervous system (peripheral ganglia and andrenal medulla) tumours	miR-17∼92	*cdkn1a*	Induction, chondrocyte differentiation	[49]
		miR-34a	*e2f3, mycn, bcl2, cdk6*	EMT	
		miR-204	*bcl2, ntrk2*	Specification and migration	[53]
		miR-193b	*mycn, mcl1, ccnd1*	Orofacial development	[51]
		miR-188	*kif1b* (predicted)		[52]
		miR-125-1	*e2f3, mcl1* (predicted)	Specification	
		miR-501	*bcl2, e2f3, cdk6, ntrk2* (predicted)		
Neurofibromatosis	Peripheral nerves and Schwann cells tumour	let-7b	*lin28b*	Chondrocyte differentiation	[75]
		miR-143	*bcl2, fgf1, igfbp5, camk1d*	Cardiac differentiation	
		miR-145	*tgfr2, apc, cmyc*	Cardiac differentiation	
		miR-135	*lzts1, lats2, ndr2, btrc*		
		miR-889	*apc*		
		miR-128	*nf1*		[76]
		miR-137	*nf1*		
		miR-103	*nf1*		

Many individual miRNAs have now been associated with aspects of NC development. One of the first studies was carried out by Gessert and colleagues [36]. They found that miR-130a, miR-219, miR-23b, miR200b, miR-96 and miR-196a are involved in eye and NC development in *Xenopus laevis*. By using a morpholino approach, they further showed that miR-130a, miR-219 and miR-23b are essential for the correct development of the eye, while the knock-down of miR-200b, miR-96 and miR-196a cause, other than eye phenotypes, craniofacial defects often associated with NC defects.

In 2013, Avellino and colleagues investigated the role of miR-204 during NC migration. They found that by modulating the expression of miR-204 in Medaka fish embryos, they were able to increase or reduce NCC migration. They also demonstrated that *Ankrd13A* is a direct target of miR-204 and an important modulator of NCC migration [37]. It is worth noting that, among the human *in-silico* predicted targets of miR-204, there is *Ankrd13C*, a paralog of *Ankrd13A*, and other genes involved in motility and extracellular matrix stability, such as the metalloprotease, *Adamts9*, the cadherins, *Cdh4* and *Cdh11*, and the collagen, *Col5A3*. If these *in-silico* targets were validated in NCCs, it would mean that miR-204 is able to regulate NC migration by targeting several key proteins involved in this process.

These results were partially confirmed by Ward and colleagues [38]. In this study *Xenopus laevis* embryonic organoids, often termed ‘animal caps’ were induced to become either NC or neural tissue by injection of Wnt1 and Noggin mRNA (to induce NC) or with Noggin alone (to induce neural tissue). The resulting induced-animal caps were then subjected to small RNA next generation sequencing and differential analysis carried out to identify miRNAs specifically expressed in NC tissue. The most abundant miRNA species detected in NC-induced animal caps were miR-219, miR-196a, miR-218-2, miR-10b, miR-204a, miR-130b/c, miR-23, and miR-24, with miR-219 as the most enriched miRNA in NC-induced animal caps, followed by miR-196a. Further experiments using luciferase assays to validate targets of miR-219 found that *Eya1* is directly targeted by this miRNA (Ward and Wheeler, unpublished results). Other enriched miRNAs were miR-301a and miR-338-3, but these were also found to be expressed in blastula stage animal caps and could be involved in maintaining pluripotency of the blastula stage ectoderm and putative NC.

Recently, we developed an efficient method to knockout miRNAs using CRISPR/Cas9. To do this, we generate two sgRNAs flanking the miRNA sequence in the genome. Introduction of these sgRNAs plus Cas9 into the embryo leads to deletion of the whole miRNA pri-RNA sequence. Using this method, we have knocked out miR-219 and miR-196a, showing clear NC phenotypes, including craniofacial and pigment abnormalities [39]. The observed phenotype for miR-219 could be due to the direct down-regulation of *Pdgfra* and *Sox6* which have been shown to be targets of miR-219 in oligodendrocyte differentiation and myelination [40].

More recent work in chick, highlighted how miR-20a, miR-200a and miR-217 contribute to NC identity by inhibiting FGF pathway on different levels, reducing the levels of *Fgf4*, *Fgf13* and *Fgfr2* in the NC region [41]. In a similar way, other miRNAs have been shown to modulate other key pathways during NC development. For example, Bhattacharya and colleagues showed how the Wnt signalling pathway is modulated by the Lin28/miR-let-7 axis. In particular, high levels of Lin28a promoted by Wnt inhibit the activity of miR-let-7. When NCCs migrate away from the Wnt source, the level of Lin28a is reduced, and this results in an increased level of miR-let-7 activity. The effect leads to the repression of the NC multipotency factors, such as *Pax3/7*, *FoxD3* and *cMyc* [42].

A number of groups have reported a role for specific miRNAs during NC differentiation [31]. For example, Steeman and colleagues have shown that the highly conserved miR-145, which is transcribed together with miR-143, plays a role during craniofacial development in zebrafish. They speculated that this might be caused by a direct interaction between miR-145 and *Sox9b* [43]. Zhao and colleagues, also working in zebrafish, have shown that miR-1 plays a role in NC development, as its knock-down produces defects during craniofacial, heart, melanocyte and iridophore development [44].

Other studies have revealed that miR-375 is up-regulated in chromaffin cells from the adrenal medulla, another NC-derived tissue involved in the synthesis of the catecholamines adrenaline and noradrenaline [45]. It has been observed that miR-375 acts as negative regulator of TH and DBH (two key enzymes involved in the synthesis of catecholamines). In particular, the authors showed that miR-375 targets *Sp1*, the regulator of TH and DBH, in response to stress [46]. Another study conducted by Shtukmaster and colleagues demonstrated that miR-124 is also expressed in developing sympathetic neurons and supports the maintenance of neuronal morphology in sympathoadrenal cells [47].

Figure 1 shows various miRNAs that have been so far identified to potentially play a role in NC specification, migration and differentiation. Future work needs to determine the specific effects of these miRNAs in NC development. In particular, at what point in NC development do they act and how are they regulated? Also, it will be necessary to validate functional and direct targets of the miRNAs involved in NC development. For example, a luciferase assay shows if there is a direct interaction between a miRNA and an mRNA under a non-physiological expression of both miRNA and mRNA, but it does not provide information about the spatial-temporal expression of those two molecules and whether they interact *in vivo*. To assess the role of a miRNA in the NC-GRN, it is necessary to investigate what factors regulates its expression and verify that the miRNA targets are expressed in the same tissue and at the same stage of development.

## miRNAs and neurocristopathies

Neurocristopathies (NCPs) are diseases that can arise due to problems occurring at any time during the development of the NC. These defects can affect a single NC-derived tissue as in albinism that only affects melanocytes, or they can be syndromic and affect several NC-derived tissues as in CHARGE syndrome, which causes coloboma, heart congenital defects and genital abnormalities [48].

MiRNAs are increasingly becoming associated with various NCPs (Table 1). Despite the fact that NCPs are among the most studied genetic diseases, the etiopathogenesis of many NCPs remains to be elucidated, and many factors involved are still to be discovered.

As mentioned before, a well characterised NCP that affects 1 in 4000 to 6000 live births is DiGeorge Syndrome (DGS). The pathology of this condition is characterised by a combination of phenotypes, including cardiac defects, abnormal facies, cleft palate and an absent or hypoplastic thymus. Other common symptoms are renal anomalies, hearing loss and skeletal abnormalities. DGS is often caused by a deletion of 22q11.2, a region that includes the gene that encodes for DGCR8, an important cofactor of DROSHA and essential for proper miRNA biogenesis [35]. The fact that loss of DGCR8 is associated with a syndromic NCP is a strong indication that the miRNA pathway plays an important role at many levels of NC-development.

In recent years, de-regulated miRNAs have been associated with different types of NCPs and NC-derived cancers (Table 1) [49–53]. Some types of cancers, in particular neuroblastoma (NB) and melanoma, are considered NCPs, as they derive from NC tissues. MiRNAs that promote tumour growth are called oncomiRs, while miRNAs known to suppress the malignancy of the tumoral mass are called anti-oncomiRs [54]. MiRNAs associated with NB aggressiveness include the cluster miR-17∼92, which contains five miRNAs (miR-17, miR-18a, miR-19a, miR-20a and miR-92). The overexpression of this cluster in NB is associated with high proliferation and invasiveness, while down-regulation reduces the invasiveness and increases apoptosis in these cells. On the other hand, miR-34a has been shown to have a protective role, as the overexpression of this miRNA induces the arrest of cell proliferation and apoptosis in NB cells. This effect might be due to the targeting of *cdkn1a* by miR-17-p, while miR-34a is shown to directly target *E2F3*, which induces cell cycle progression [49]. MiRNAs have also been associated with melanoma. For example, miR-21 is considered an oncomiR, as its expression is often up-regulated in melanoma cells. Its actions involve the inhibition of cell differentiation and apoptosis. Moreover, knock-down of this miRNA in melanoma cells induces apoptosis and enhances the effectiveness of chemotherapy and radiotherapy. Also in this case, other miRNAs have been shown to have oncosupressor activity. MiR-32 is one of these as it promotes the arrest of melanoma growth by inhibiting the expression of MCL-1 and, by doing so, the PI3K-AKT-mTOR pathways [50].

Given the increasing number of findings that associate an altered expression of miRNAs and cancer, research groups are starting to give particular attention to regions of DNA harbouring non-translated genes (miRNAs, lncRNAs, piRNAs) and the non-coding regions of mRNAs which are, in fact, important post-transcriptional regulators via interaction with RNA-binding proteins and miRNAs [55,56]. This trend is providing insights into additional facets of gene regulation, mechanisms of development and mechanisms that lead to pathological conditions.

As an example, Bachetti and colleagues (2021) made an association between the miRNA-mediated regulation of *phox2B* and a pathological condition, congenital central hypoventilation syndrome (CCHS), an NCP that affects the correct development of the CNS and which can cause sudden infant death (SUID) via hypoxic crisis which occurs during sleep [57]. They observed a point mutation in the 3′ UTR of *phox2B* [58]. This generates a potential new binding site for miR-204, which is already known to target *phox2b* mRNA in NB cells [59,60]. They speculated that the generation of this new binding site for miR-204 is the cause of the down-regulation of *phox2b* expression, and that could contribute to the occurrence of SUID [58].

Another NCP that has recently been associated with miRNAs is Hirschsprung Disease (HD), a condition characterised by absence of enteric ganglia. This condition impairs passing stool and can lead to a series of signs such as swollen belly, vomiting, chronic constipation, and fatigue [61]. In 2016, a differential miRNA expression analysis on colon tissue from HD patients was carried out. 168 differentially (up-regulated and down-regulated) expressed miRNAs were identified between Hirschsprung and healthy tissues [62]. In recent years, a number of miRNAs has been associated with HD, including miR-100, miR-206, miR-214 and miR-483. For example, a point mutation in the miR-100 gene has been shown to increase HD susceptibility in a southern China population [63].

These studies are leading the way for a new concept underpinning the diagnosis of rare diseases, in which clinicians analyse regions of the genome producing protein coding mRNAs and/or non-coding RNAs to make predictions. In the future, this approach might be used to treat these conditions before the appearance of symptoms, providing the families of these patients with an alternative that could actually cure the disease [64,65].

## Perspectives

- The GRN underlying the induction, migration and specification of the NC is under constant revision.
- Further studies that focus on the role of non-coding RNA species, such as miRNAs, during NC development are fundamental in order to increase our knowledge of the NC-GRN.
- Understanding the role of miRNAs in NC-development can provide clinicians with more powerful tools for the diagnosis of NCPs and other rare diseases.

## Open Access

Open access for this article was enabled by the participation of University of East Anglia in an all-inclusive *Read & Publish* agreement with Portland Press and the Biochemical Society under a transformative agreement with JISC.

## Abbreviations

BMP: Bone Morphogenic Protein
CCHS: Congenital Central Hypoventilation Syndrome
DGS: DiGeorge syndrome
EMT: Epithelial to Mesenchymal Transition
FGF: Fibroblast Growth Factor
GRN: Gene Regulatory Network
lncRNA: long non-coding RNA
miRNA: microRNA
MMP: Matrix Metalloproteinases
mRNA: messenger RNA
NB: Neuroblastoma
NC: Neural Crest
NCCs: Neural Crest Cells
NCP: Neurocristopathy
NPB: Neural Plate Border
ORF: Open Reading Frame
piRNA: Piwi-interacting RNA
RISC: RNA Inducing Silencing Complex
sgRNA: short guide RNA
SUID: Sudden and Unexpected Infant Death
UTR: Untranslated Region
